# Multi-Day EMG-Based Knee Joint Torque Estimation Using Hybrid Neuromusculoskeletal Modelling and Convolutional Neural Networks

**DOI:** 10.3389/frobt.2022.869476

**Published:** 2022-04-25

**Authors:** Robert V. Schulte, Marijke Zondag, Jaap H. Buurke, Erik C. Prinsen

**Affiliations:** ^1^ Roessingh Research and Development, Enschede, Netherlands; ^2^ Department of Biomedical Signals and Systems, University of Twente, Enschede, Netherlands; ^3^ Department of Biomechanical Engineering, University of Twente, Enschede, Netherlands

**Keywords:** myoelectric control, neuromuscular modelling, lower limb, torque estimation, motor intent recognition, machine learning

## Abstract

Proportional control using surface electromyography (EMG) enables more intuitive control of a transfemoral prosthesis. However, EMG is a noisy signal which can vary over time, giving rise to the question what approach for knee torque estimation is most suitable for multi-day control. In this study we compared three different modelling frameworks to estimate knee torque in non-weight-bearing situations. The first model contained a convolutional neural network (CNN) which mapped EMG to knee torque directly. The second used a neuromusculoskeletal model (NMS) which used EMG, muscle tendon unit lengths and moment arms to compute knee torque. The third model (Hybrid) used a CNN to map EMG to specific muscle activation, which was used together with NMS components to compute knee torque. Multi-day measurements were conducted on ten able-bodied participants who performed non-weight bearing activities. CNN had the best performance in general and on each day (Normalized Root Mean Squared Error (NRMSE) 9.2 ± 4.4%). The Hybrid model (NRMSE 12.4 ± 3.4%) was able to outperform NMS (NRMSE 14.3 ± 4.2%). The NMS model showed no significant difference between measurement days. The CNN model and Hybrid models had significant performance differences between the first day and all other days. CNNs are suited for multi-day torque estimation in terms of error rate, outperforming the other two model types. NMS was the only model type which was robust over all days. This study investigated the behavior of three model types over multiple days, giving insight in the most suited modelling approach for multi-day torque estimation to be used in prosthetic control.

## 1 Introduction

Amputees regain functional abilities by learning to use a prosthesis (Windrich et al., 2016). Most prostheses are passive and are adequate for most walking scenarios in daily life. However, these passive prostheses are not capable of producing power. Therefore, the use of these prostheses in daily life is limited by an amputee’s strength and capacity for gait adaptations. This makes activities such as stair climbing challenging (Dabiri et al., 2010; Crowe et al., 2019). Actuated or powered prostheses have the potential to overcome these limitations by providing net power (Windrich et al., 2016). Commercially available powered prostheses use state-machines to provide control in predefined states. The downside of this approach is that the control is limited in intuitiveness and the user is not able to use the prosthesis outside of predefined states. Direct control over the knee joint might result in a more intuitive control of the prosthesis, which can be beneficial for the user during various tasks of daily living. It can be beneficial to apply direct control within a discrete state, for example to reposition the prosthesis while sitting or to control the speed of a motion. Surface electromyography (EMG) can play a large role in creating intuitive control, as EMG can be measured 138 ms before the onset of movement (Wentink et al., 2014). Other sensor information related to movement, such as acceleration, will always lag behind the intended motion and will result in a delay within the control scheme. EMG does not suffer from this delay and translating EMG into joint torque is therefore a promising method to realize voluntary control.

Machine learning can be a viable tool for joint torque prediction. Neural networks are a type of machine learning algorithms that are trained to find the underlying relation between input and output variables. Several studies used neural networks for the prediction of knee flexion angles or torques in able-bodied subjects (Liu et al., 2018; Huang et al., 2019; Saranya et al., 2019; Wang G. et al., 2019; Deng et al., 2020; Gautam et al., 2020). Huang et al. (2019) proposed a deep-recurrent neural network for prediction of knee joint angles in real-time. The model used EMG signals together with inertial data from different activities and reported a root mean squared error of 2.93° over a range of approximately 60° (4.9% error). Gautam et al. (2020) used a Long-term Recurrent Convolution Network to classify movements and predict their corresponding knee joint angles, based on EMG. They reported an average mean absolute error of 8.1% in the knee angle prediction of healthy subjects. Zhang et al. (2020) developed an artificial neural network for the prediction of ankle torque from EMG. Root mean squared error (RMSE) values ranging within 0.01 and 0.10 Nm/kg were found for ankle plantar- and dorsiflexion on a range of approximately 1.5 Nm/kg (0.7–6.7% error). All these studies show low error rates, which indicates that machine learning can be a valuable tool in predicting knee torque or knee angle. However, current machine learning decoders might produce unrealistic estimates in conditions they are not trained in as the algorithm are not limited by physical bounds (Sartori et al., 2018). Next to this, the reliability of these methods depend on correct electrode placement and are thus sensitive to changes in conditions. Futhermore, the amount of training data is also usually limited relative to the complexity of the models, which makes it difficult to obtain a satisfactory generalization performance (Wang W. et al., 2019). The robustness to EMG electrode placement, differences in EMG signals (quality), activity performance and performance over days is thus questionable and needs futher investigation.

One potential way to improve robustness, is the use of an NMS model. Neuromusculoskeletal (NMS) modelling was designed to gain insight in the underlying process of biomechanical movement to characterize motor function and how it alters with pathology (Durandau et al., 2019). An NMS model consists of multiple components that model this underlying process. It uses EMG and muscle characteristics from a musculoskeletal model to predict specific muscle activations with corresponding forces and resulting joint torques. An NMS model can provide system robustness since any joint moment estimate must always exist within the musculoskeletal model operational space and be therefore physiologically plausible (Sartori et al., 2018). Another benefit of using an NMS model is that it provides insight in the underlying process of biomechanical movement, whereas machine learning does not. Several studies used an NMS model to predict joint torque (Wang W. et al., 2019; Sartori et al., 2018; Durandau et al., 2019, 2018; Sartori et al., 2012, 2016; Kapelner et al., 2020). Sartori et al. (2012) developed a control scheme to control a wrist-hand prosthesis by real-time neuromusculoskeletal modelling. Joint torque was predicted using EMG and prosthesis angles as input and translated into low-level control of the prosthesis. They executed a virtual reaching test in which subjects reached targets using linear trajectories, thereby successfully actuating a single DOF at a time with high precision. Path similarity was always accomplished with *R* ^2^ > 0.98 across all targets and subjects. Durandau et al. (2019) predicted lower limb exoskeleton support torque, using EMG and joint angles as input to an NMS model. The RMSE for the knee joint control, inside exoskeleton conditions, were 4.06 ± 2.55° for low gain and 4.58 ± 2.61° for high gain over a range of approximately 40° (approximately 10–11% error). Correlation coefficients of both conditions between prediction and reference torque were 0.90 ± 0.16 and 0.92 ± 0.07 respectively. Zhang et al. (2020) used an NMS model to predict ankle joint torque with RMSEs ranging from 0.04 to 0.18 Nm/kg for ankle plantar- and dorsiflexion on a range of approximately 1.5 Nm/kg (2–12% error). Downsides of NMS modelling workflow are that it can be complex and difficult to work with. Furthermore, the mapping of EMG to muscle activation is based on models that are difficult to validate because activations cannot be measured directly.

Since both machine learning and NMS models show several shortcomings, the question arises if combining these two methods into a hybrid version will provide better joint torque predictions. Machine learning can decrease computational demands in physics-based modelling. It can be used for feature extraction from measures of muscle activation and to synthesize missing data (Saxby et al., 2020). Cimolato et al. (2020) developed a machine learning driven NMS model to predict lower limb joint torque from EMG and inertial data to control a lower limb prosthesis during regular gait. They used a Gaussian Mixture Regressor to generate a complete set of EMG signals, starting from the supposed residual subset of available EMGs. These EMG signals were then used as input for a calibrated NMS model to predict joint torque, which resulted in an average normalized root mean squared error (NRMSE) of 24.0 ± 11.0%. Xu et al. (2020) developed an EMG-based elbow joint torque estimation strategy using a Hill-Type Muscle Model and a neural network. The neural network was used to estimate muscle activation which was used as input for the Hill-Type model. System identification from EMG signals was used to estimate the elbow angle. The average RMSE over trials was 1.45 Nm on a range of 25 Nm (5.8% error). Only one subject was used and three trials were conducted on the same day. Although these studies show lower accuracies than machine learning or neuromuscular modelling, the idea of combining neuromuscular modelling with machine learning still holds promise.

In the described studies we have seen that machine learning, neuromuscular modelling or a combination of both are promising approaches for knee joint torque estimation. Despite these positive results, to our knowledge, no commercial product is available that uses machine learning, NMS or a hybrid approach for direct control of a prosthetic knee. One of the reasons is that prostheses need to be used in daily life and need to function everyday in a reliable manner, being robust to different environmental conditions, such as electrode placement and skin conditions, but little is known of the behavior of these approaches over multiple days.

The goal of this study was to compare three model types to predict knee joint torque from EMG, to be used for multi-day control of a transfemoral prosthesis in non-weight bearing situations. In this work we used a convolutional neural network (CNN) using recurrent layers to predict knee joint torque, as this architecture is useful for biomedical time series modeling (Gautam et al., 2020). To our knowledge, CNNs have not been applied before for knee joint torque prediction. We evaluated the models over multiple days, to see their robustness when applied in a multi-day setting. Our expectations are threefold. Firstly, CNN models will show excellent performance on the training day, but performance will decrease on subsequent days. Secondly, NMS models will be robust from day to day, hence outperforming machine learning on subsequent days, but would not reach as high performance as CNN models on the first day. Finally, a combination of these two model types in a hybrid model, is expected to incorporate the best of both worlds: reaching as high performance in knee joint torque prediction as CNN models, while being as robust as NMS models.

## 2 Methods

### 2.1 Experimental Data

Data were collected at the Roessingh Research and Development (RRD), in Enschede the Netherlands, as part of the MyLeg project. Ten able-bodied subjects (sex: 4m, 6f, age: 23.7 ± 2.4 years, length: 173.8 ± 6.4 cm, weight: 71.0 ± 8.8 kg), participated in this study. The protocol was reviewed and approved by Medical research Ethics Committees United (MEC-U) Nieuwegein, the Netherlands, with trial number NL67247.044.18. The participants provided their written informed consent before inclusion in the study.

Bipolar EMG was recorded from four muscles on both legs: rectus femoris (RF), vastus lateralis (VL), biceps femoris (BF) and semitendinosus (ST). All EMG electrodes were placed according to SENIAM guidelines (Hermens et al., 2000). The data was acquired using the Cometa Wave electrodes at a sampling frequency of 2000 Hz. The RF, VL, BF and ST were included for this study since the intended application is for a prosthetic knee and these muscles extend over the knee. The RF and VL contribute to knee extension, whereas the BF and ST contribute to knee flexion. Kinematics were measured using eight IMUs (Xsens Link, Enschede, Netherlands), placed on the sternum, pelvis and bilaterally on the thigh, shank and foot of the subject. Data was recorded with a sampling frequency of 240 Hz. Joint angles were reconstructed from Xsens MVN software. Only knee joint angles in the sagittal plane were used in this study. EMG and kinematics were time synchronized and resampled to 1000 Hz.

**FIGURE 1:**
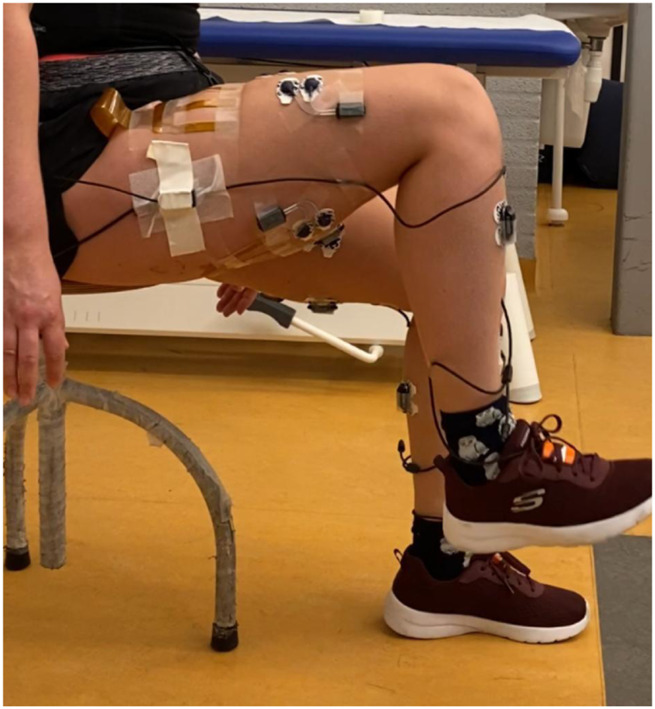
Non-weight-bearing setup: subject is seated on a stool with one foot slightly lifted off the ground.

Each subject was measured four times: three measurements were conducted on three subsequent days on day 1, 2 and 3 and the last measurement was four days later on day 7. The subjects were measured during the same time slot on each day. Before each measurement the EMG of the maximal voluntary contraction (MVC) of each muscle was measured to normalize EMG. The subjects were asked to perform a trial containing a set of activities, including level-ground walking, stair ascent/descent, ramp ascent/descent, sit-stand motions and non-weight-bearing activities on a stool. Only the non-weight-bearing activities were used in this study as these form the starting point for safely implementing a torque estimation strategy. The subject had to sit on a stool and lift one leg off the ground (knee approximately 90°), as can be seen in Figure 1. Then, the subject had to fully extend the knee while keeping the foot perpendicular to the lower leg. After, the subject performed maximal plantarflexion of the ankle, followed by maximal dorsiflexion. The knee was then brought back to a knee angle of approximately 90°. Then, only knee extension and flexion needed to be performed. Lastly, only ankle plantar- and dorsiflexion needed to be performed while keeping the knee angle at 90°. After, the foot was set down on the ground and the routine was repeated with the other leg. The trial in which this routine was included, was performed twenty times. Then, the routine was slightly changed for another twenty trials: the subject had to first perform ankle plantar- and dorsiflexion, then the combination of both knee and ankle extension and flexion, and finish off with only knee extension and flexion. Although ankle movements were performed, only the knee joint torque was of interest for this study. In total forty trials were performed.

### 2.2 Data Pre-Processing

The smoothed rectified envelope (SRE) of the raw EMG data of the four muscles (RF, VL, BF, ST) was obtained by high-pass filtering at 20 Hz, rectifying and low-pass filtering at 6 Hz. All filters were zero-lag 2nd order butterworth filters. The SREs were normalized by values obtained from the MVCs. Torque reference data was obtained using OpenSim 4.1, an open source software kit to develop musculoskeletal models and make dynamic simulations (Seth et al., 2018). First, the OpenSim lower extremity and torso model Gait2392 was scaled using subject body measures to create a subject-specific model. knee joint torque was obtained using the measured kinematics and the Inverse Dynamics tool, without using external ground reaction forces. Next, the torque was low-pass filtered using a 2nd order zero-lag butterworth filter with a cutoff frequency of 1 Hz. The resulting knee joint torque was used to train and calibrate the developed models in this study. Muscle moment arms and muscle tendon unit lengths of the MTUs were extracted from the scaled model. These parameters were used in the NMS and Hybrid models.

### 2.3 Convolutional Neural Network

The first data pipeline contained a machine learning model, consisting of a convolutional neural network (CNN) which maps the windowed SREs to knee joint torque. SREs were windowed using a window of 128 ms and a stride of 16 ms. Corresponding knee joint torques were windowed as well and the average torque value over a window was used as reference.

The CNN extracts local features from input images, using a convolutional layer with a local receptive field. It then uses layers with certain activation functions to map these features to the desired output. In this work a Long Short Term Memory (LSTM) layer was added to the CNN. An LSTM is an artificial recurrent neural network architecture (Saranya et al., 2019), which has internal mechanisms that can regulate the flow of information and learn which data in a sequence is important to keep or discard. For this study, no fixed CNN model architecture was used. The model architecture was determined using Bayesian optimization by minimizing the loss between predicted and reference torque for all subjects on a training set of the first measurement day. The loss function used in this study was the mean squared error over the correlation coefficient:

$$loss=\frac{\frac{1}{n}\sum_{i=1}^{n}{\left(Y_{i}-{\hat{Y}}_{i}\right)}^{2}}{r^{2}}$$ (1)

$$r=\left\{\begin{array}{cc} corr\left(Y,\hat{Y}\right)\; & >0\\ 1\text{e}-2\; & \text{otherwise} \end{array}\right.$$ (2)

Herein is *Y* the reference torque and $\hat{Y}$ the estimated toruqe. Correlation values less that zero were set to 1e-2 to avoid numerical instability. By using the correlation coefficient in combination with the mean squared error the shape of the curve of the torque prediction could also be taken into account (correlation coefficient), while still penalizing large deviations (mean squared error). Each model architecture existed of a variable number of convolutional layers with relu activation, an LSTM layer, a drop out layer with variable dropout rate and a final dense layer with linear activation. The used hyperparameters and corresponding search space were: the number of convolutional layers {1,2,3,4}, the number of filters in the convolutional layer {16, 32, 64}, the convolutional kernel size {3,5,7}, the number of LSTM units {16, 32, 64}, whether to use batch normalization after each layer {True, False}, and the drop out rate {0.01,0.1,0.2,0.3,0.4}. The optimizer was Adam with a learning rate of 0.001. The optimization routine ran for 200 iterations. During each iteration a model was trained with a maximum of 100 epochs. Training was stopped early if the loss did not reduce for 5 epochs. Overview of the fully-optimized architecture is shown in the results section.

After hyperparameter optimization the optimized model architecture was used for all subjects. The model was trained per leg of each subject, to make the model subject specific. The predicted torque was low-pass filtered with a second order zero-lag low-pass filter and a cut-off frequency of 1 Hz to obtain a smooth prediction.

### 2.4 NMS Model

The second data pipeline used a neuromusculoskeletal model (NMS model) which maps EMG to knee joint torque. In this work we implemented Hill-type muscle models as described by Thelen (Thelen, 2003; Miller et al., 2018). The NMS model consists of four parts:

#### 2.4.1 Neural Activation Dynamics

The muscle activity *a* was determined by a first order differential equation (Thelen, 2003) and a non-linear transfer function (Sartori et al., 2012).

$$\dot{u}=\frac{emg-u}{\tau_{a}\left(u,emg\right)}$$ (3)

$$a=\frac{e^{Au}-1}{e^{A}-1}$$ (4)

Herein is *emg* the SRE of the muscle of interest, *u* the neural activation and *a* the muscle activation. *A* determines the shape of the transfer function. *τ*_*a*_ is a time constant that varies with activation level:

$$\tau_{a}\left(u,emg\right)=\left\{\begin{array}{cc} \tau_{act}\left(0.5+1.5u\right)\; & emg>u\\ \tau_{deact}/\left(0.5+1.5u\right)\; & \text{otherwise} \end{array}\right.$$ (5)

The time constant for activation *τ*_*act*_ and the time constant for deactivation *τ*_*deact*_ were parameters that were optimized.

#### 2.4.2 Contraction Dynamics

The muscle fiber force is described by Eq. 6.

$$F_{m}=F_{max0}\left(af_{v}\left({\dot{l}}_{m}\right)f_{a}\left(l_{m}\right)+f_{p}\left(l_{m}\right)+d_{m}{\dot{l}}_{m}\right)\cos\alpha \left(l_{m}\right)$$ (6)

Herein is *F*_*m*_ the muscle fiber force, *F*_*max*0_ the maximal isometric force, *a* the muscle activity, *f*_*v*_ the force-velocity relationship, *f*_*a*_ the active force-length relationship, *f*_*p*_ the passive force-length relationship, *d*_*m*_ the velocity damping factor and *α* the pennation angle. All relationships depend on the normalized muscle fiber length *l*_*m*_ and its derivative the normalized fiber velocity ${\dot{l}}_{m}$ . The fiber length was normalized by the optimal fiber length at maximal isometric force, *l*_*opt*0_. The normalized fiber length was adjusted for activation level in *f*_*a*_ as described by Lloyd and Besier (Lloyd and Besier, 2003; Miller et al., 2018). The tendon force *F*_*t*_ was depended on the tendon strain *ϵ*_*t*_ Eq. 8 via the tendon length *l*_*t*_ Eq. 7 as described by Thelen (2003). The tendon length depended on the muscle-tendon length *l*_*mt*_ which was determined using the scaled Gait2392 model using the corresponding joint angles.

$$l_{t}=l_{mt}-l_{m}\cos\alpha \left(l_{m}\right)$$ (7)

$$\epsilon_{t}=\frac{l_{t}-l_{slack}}{l_{slack}}$$ (8)

The normalized fiber length *l*_*m*_ was determined by finding the root of the equilibrium Eq. 9 using sequential least squares programming.

$$|F_{t}-F_{m}|=0$$ (9)

#### 2.4.3 Joint Mechanics

The muscle force $F_{m_{j}}$ was multiplied with corresponding moment arm *r*_*j*_ to obtain the torque contribution of muscle *j*. The knee joint torque was obtained by summing all the muscle torque contributions. The moment arms *r* were determined using the scaled Gait2392 model using the corresponding joint angle.

$$\sum F_{m_{j}}r_{j}$$ (10)

#### 2.4.4 Optimization Routine

For each muscle made subject specific by optimizing hyperparameters. These hyperparameters and corresponding search spaces for each muscle were: the activation and deactivation time constants *τ*_*act*_ {10–80, step = 0.1} and *τ*_*deact*_{10–80, step = 0.1}, the shape factor of the non-linear transfer function *A* {-3 to −0.01, step = 0.001}, the isometric strength coefficient *c*_*str*_ {0.5–1.5, step = 0.05}, the slack coefficient *c*_*slack*_ {0.85–1.15, step = 0.05}, and the optimal fiber length coefficient *c*_*opt*0_ {0.85–1.15, step = 0.05}. The latter three coefficients adjust parameters from the contraction dynamics:

$$\begin{array}{ll} {\hat{F}}_{max0} & =c_{str}F_{max0}\\ {\hat{l}}_{slack} & =c_{slack}l_{slack}\\ {\hat{l}}_{opt0} & =c_{opt0}l_{opt0} \end{array}$$

A Bayesian optimization routine was used to find the optimal hyperparameters using the same loss function as seen in Eq. 1. The optimization routine ran for 2000 iterations per leg per subject on the training set of the first day. The predicted torque was low-pass filtered with a second order zero-lag low-pass filter and a cut-off frequency of 1 Hz to obtain a smooth prediction.

### 2.5 Hybrid Model

The third data pipeline contained a Hybrid model which consists out of parts of both the CNN and NMS model. A CNN was used to replace the activation component of the NMS model described by Eqs 3–5. The CNN model architecture was built from the hyperparameters found by the optimized ML model. The activation of the last dense layer of the ML model was changed from a linear to a sigmoid function, to limit the output activation per muscle between zero and one. Hereafter a simplified force component of the NMS model was used, which is described by Eq. 11. The difference with Eq. 6 is the removal of the passive force component to simplify the model. Next to that, only two coefficients were optimized, *c*_*str*_ and *c*_*slack*_ to further simply the model. This was necessary as the optimization routine would be too complex otherwise and no adequate solution would be reached.

$$F_{m}=F_{max0}\left(af_{v}\left({\dot{l}}_{m}\right)f_{a}\left(l_{m}\right)+d_{m}{\dot{l}}_{m}\right)\cos\alpha \left(l_{m}\right)$$ (11)

The training process was a combination of the CNN and NMS calibration. First the NMS parameters were adjusted based on the SREs without any activation dynamics for 500 iterations using a Bayesian optimization routine as described before. Hereafter the CNN part of the model was used to estimate the activation based on the windowed SREs and trained for 10 epochs. After this, the NMS parameters were adjusted again, this time using the CNN-based activations as input, again for 500 iterations and then the CNN was trained again for 10 epochs. Hereafter the NMS parameters and CNN-parameters were optimized for a final time. The loss function was the same as for CNN and NMS models, see Eq. 1.

Windowed SREs were used as input and mapped onto muscle activations by the CNN. These predicted activations were used as input for the remaining NMS model. Windowed muscle-tendon lengths and moment arms were used in this remaining NMS model. The predicted torque was low-pass filtered with a second order zero-lag low-pass filter and a cut-off frequency of 1 Hz to obtain a smooth prediction.

### 2.6 Data Evaluation

Three different models were trained for each leg from each subject. A fixed train/test split of 80/20% was made on data from the first measurement day. All models were trained and validated on the training set (80%), in which a shuffled train/validate split of 80/20% was made. All models were tested on the test set (20%) and all data from remaining measurement days. This separation in data was made for the intended application: it would be ideal to train a model on just one day and to have it perform well on every other day. With this method, robustness of all models against varying circumstances could be tested.

The performance metric used for this study was the normalized root mean squared error (NRMSE (%)), calculated by Eq. 12. $\hat{x}$ denotes the predicted datapoint, with *x* as reference, *N* equals the total number of datapoints of which *t* indicates one specific datapoint. max(*x*_*t*_) and min(*x*_*t*_) are the highest respectively lowest torque reference value of all trials of one leg and are used to normalize the data.

$$NRMSE\left(\%\right)=\frac{\sqrt{\frac{1}{N}\sum_{t=0}^{N}{\left({\hat{x}}_{t}-x_{t}\right)}^{2}}}{max\left(x_{t}\right)-min\left(x_{t}\right)}\cdot 100\%$$ (12)

Robustness was indicated by not-significant differences at the 0.05 significance level, in mean NRMSE values on different measurement days.

### 2.7 Statistical Analysis

To analyze the performance of the models, NRMSEs of all legs, of all trials from separate days were computed with each model. A Mixed Model analysis with Šidák correction was used to determine significance (*α* = 0.05) between the general performance of each model compared to one another and the performance of each model from day to day, compared by day and by model. A log transformation was performed on the NRMSE values to get normally distributed data.

### 2.8 Software

All models were built in Python 3.9. The CNNs were built using Tensorflow 2.4 (Abadi et al., 2015) and Bayesian hyperparameter optimization was done using Optuna 2.0 (Akiba et al., 2019). The Mixed Model analysis was performed using IBM SPSS Statistics Version 27.

## 3 Results

**FIGURE 2:**
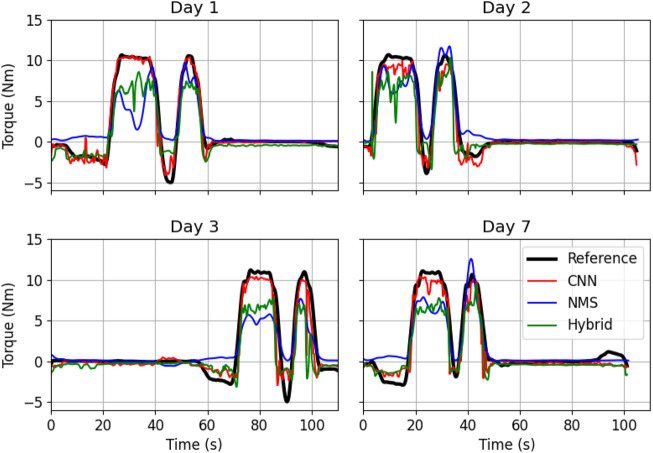
Example of torque estimation on the test set of one subject by the three different models over the four measurement days. In black the reference torque, in red the predicted torque by the CNN model, in blue by the NMS model and in green by the Hybrid model.

CNN hyperparameter optimization led to the following architecture: three convolutional layers, with 32 filters and a kernel size of 7 and an LSTM layer with 64 filters. Batch normalization was applied between each layer and a dropout rate of 0.1 was used. For the CNN part of the Hybrid version a similar architecture was used. See also Table 1. An example of the torque predictions by the three different type of models can be seen in Figure 2.

**TABLE 1:** Optimized architecture of the CNN model and the CNN part of the hybrid model.

Layer	Size	No. Parameters	Notes
Batch Normalization		128	
Convolutional layer 1D	32	928	kernelsize 7, relu activation
Batch Normalization		128	
Convolutional layer 1D	32	7,200	kernelsize 7, relu activation
Batch Normalization		128	
Convolutional layer 1D	32	7,200	kernelsize 7, relu activation
Batch Normalization		128	
LSTM	64	24,832	tanh activation, sigmoid recurrent activation
Batch Normalization		256	
Dropout			rate 0.1
Fully connected layer	1 or 4	65 or 260	linear (CNN) or softmax (Hybrid) activation

### 3.1 Model Comparison

**FIGURE 3:**
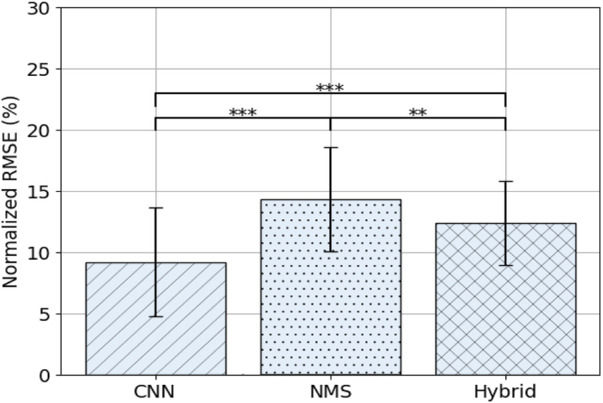
Average NRMSE (± SD) per model over all days. Asterisks indicate significance level: *p* < 0.05 = *, *p* < 0.01 = **, *p* < 0.001 = ***.

**FIGURE 4:**
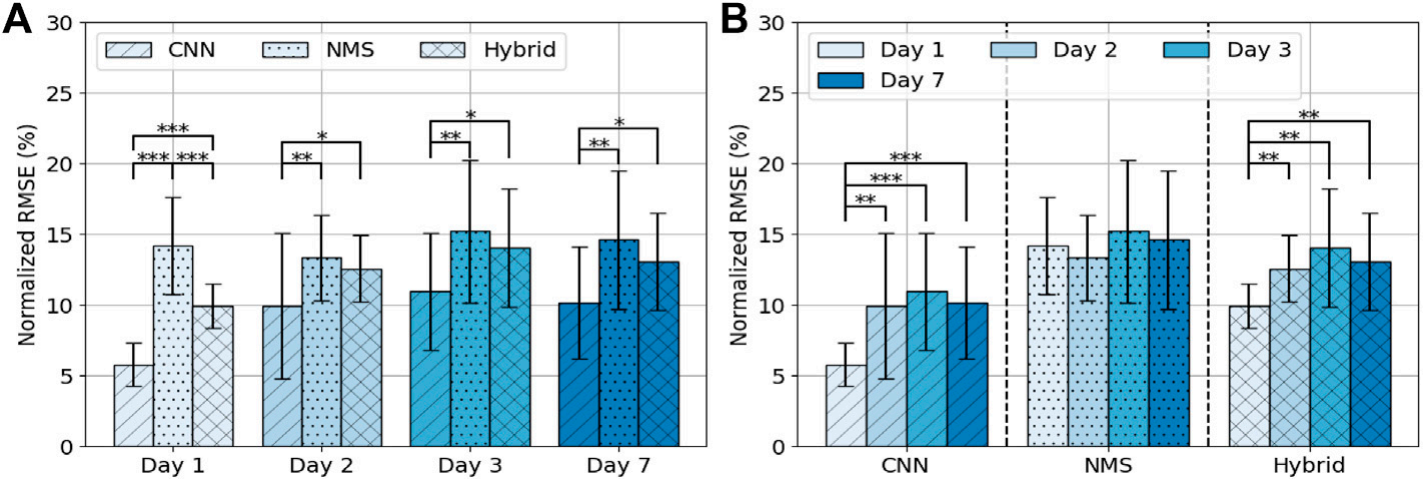
Average NRMSE (± SD) per day per model **(A)** and per model per day **(B)**. Asterisks indicate significance level: *p* < 0.05 = *, *p* < 0.01 = **, *p* < 0.001 = ***.

Overall results are shown in Figure 3 and the comparison of models over days is shown in Figure 4A. Average NRMSE was 9.2 ± 4.4% for the CNN model, 14.3 ± 4.2% for the NMS model and 12.4 ± 3.4% for the Hybrid model. The CNN model outperformed the NMS and Hybrid models overall (resp. p < 1e-4, *p* < 1e-4), on day 1 (resp. p < 1e-4, *p* < 1e-4), day 2 (resp. *p* = 0.007, *p* = 0.019), day 3 (resp. *p* = 0.004, *p* = 0.029) and on day 7 (resp. *p* = 0.001, *p* = 0.015). The Hybrid model outperformed the NMS model overall (*p* = 0.001) and on day 1 (*p* = 0.001).

### 3.2 Model Performance Over Days

Performance of the models over days is shown in Figure 4B. For the CNN the error was 5.8 ± 1.6% on day 1, 10.0 ± 5.2% on day 2, 11.0 ± 4.1% on day 3 and 10.2 ± 3.9% on day 7. The error on day 1 differed significantly from the other 3 days (resp. p < 1e-4, *p* < 1e-4, *p* < 1e-4). No significant difference was observed between day 2, 3 and 7. For NMS the error was 14.2 ± 3.4% on day 1, 13.4 ± 3.0% on day 2, 15.2 ± 5.1% on day 3 and 14.6 ± 4.9% on day 7. No significant difference was observed between day 1, 2, 3 and 7. For Hybrid the error was 10.0 ± 1.6% on day 1, 12.6 ± 2.4% on day 2, 14.1 ± 4.2% on day 3 and 13.1 ± 3.5% on day 7. The error on day 1 differed significantly from the other 3 days (resp. *p* = 0.002, *p* = 0.001, *p* = 0.004). No significant difference was observed between day 2, 3 and 7.

## 4 Discussion

The main goal of this study was to find the most suitable model to predict knee joint torque from EMG, to be used for multi-day control of a prosthetic knee joint in non-weight bearing situations. Three different models were developed and validated on both legs of ten able-bodied subjects, using multi-day measurements. The CNN model had the overall lowest prediction error (9.2 ± 4.4%) and performed significantly better than NMS and Hybrid on all days. To the best of our knowledge, our convolutional neural network was the first of its kind able to successfully predict knee joint torque from EMG input data. Next to that, we implemented state-of-the-art NMS models which showed robustness from day to day. We also successfully combined a convolutional neural network and a Hill-type muscle model to create a Hybrid model, which is also first of its kind. This Hybrid model was able to outperform NMS. This study indicates that convolutional neural networks are a suited approach to be used in knee joint torque estimation over multiple days.

The CNN showed the lowest error on the training day and had an increased error on subsequent days, which was in line with our expectations. However, the increase in error did not continue after day 2 and results showed that the CNN was robust over the other three days, showing no significant differences between day 2, 3 and 7. This is not completely in line with expectations from related work. Convolutional neural networks are known to have robustness issues (Uličnỳ et al., 2016; Ghosh et al., 2018; Arcaini et al., 2020). For example, if electrodes are placed differently on day 2 and MVC values differ from day to day, the EMG envelope images that are created can differ too much from day 1 for the CNN to make a good prediction. This may explain the significant differences between day 1 and all other measurement days. However, our findings show no significant differences between day 2, 3 or 7, thus being robust over days, which contradicts literature. A possible explanation is that the variance in EMG envelopes, and thus the input data for the CNN, within a subject is small enough to be handled by the CNN. Another explanation is that the output of the CNN used in this study is low-pass filtered to smooth any outliers, improving the NRMSE and robustness. Next to that, related work suggested that a black-box machine learning method could predict torque values outside of a physiologically plausible space (Sartori et al., 2018). We were able to develop a CNN model with low-pass filter, which was able to predict torques within a small range of the reference value. This study showed that a CNN model is suited for torque estimation within plausible ranges.

Compared to related work, our study shows similar results for the CNN model. To our knowledge no studies into the performance of knee joint angle or torque prediction over multiple days were conducted, thus only the first day performances could be compared. We observed an average error rate of 5.8 ± 1.6% on day 1, which is comparable with related work. Huang et al. reported an average error of 4.9% and Gautam et al. reported an average error of 8.1% for knee joint angle prediction. Zhang et al. reported an average error of 0.7–6.7% for ankle torque prediction.

The NMS model did not differ significantly over days as expected and is the only model that met our definition of robustness. It was expected that robustness was shown over every measurement day (Durandau et al., 2018; Sartori et al., 2018). Related work showed the model’s ability to predict torque in untrained activities, thereby proving its robustness by their definition (Durandau et al., 2018). Our definition of robustness is different since it refers to time and not the model’s ability to predict untrained activities. To our knowledge, little related work investigated robustness from day to day. Just one study calibrated the NMS model on one day and tested the model the day after, but did not test for between-days variance (Sartori et al., 2018). This study showed that NMS is also robust over multiple days. The error observed on day 2, 3 and 7 by the NMS model was significantly higher than the error of the CNN model, which was not expected. Next to that, the average NRMSE of 14.3 ± 4.2% was slightly higher than observed in related work. Durandau et al. reported an average error of 10–11% and Zhang et al. reported an average error of 2–12%. It could be that the model was too complex for the task that was performed. Furthermore, the activities performed in this study find the physiological boundaries of the knee angle and thus of the muscle parameters, which might cause this model to perform less compared to findings in literature.

The performance of the Hybrid model was midway between the performance of the CNN model and NMS. It was not as robust as NMS and did not reach as high performance as the CNN model, thus it did not meet our expectation. Similar robustness behaviour was found compared to the CNN model: robustness was found over day 2, 3 and 7. This can be explained by the fact that Hybrid and CNN models both make use of a CNN, of which its robustness was explained before. The error overall and on day 1 was significantly lower than that of NMS, thus combining NMS with a CNN can lead to better performing models. A possible explanation is that the muscle activation computed by the CNN is more accurate than the muscle activation calculated by the activation component of the NMS model. The Hybrid model thus uses the best features of both the CNN model and NMS: the CNN is used to find a better relation between EMG and muscle activation, and the NMS components provide information about the underlying process of biomechanical movement.

Compared to related work of Hybrid modelling, we found an average NRMSE of 10.0 ± 1.6% on day 1. As no multi-day studies into knee joint angle or torque prediction based on EMG were performed to our knowledge, we cannot compare the results of the other days to related work. Cimolato et al. (2020) found an average NRMSE of 24.0% with their Hybrid model and Xu et al. (2020) found an average RMSE of 5.8% with their Hybrid approach. The application of Xu et al. was on the upper limb which is different than our application in the lower limb. This might explain the difference in error rate compared with our study as the system that needs to be modelled around the elbow is less complex than around the knee. Our Hybrid approach has an error rate which falls between these two studies.

The findings of this study are promising for the use in a transfemoral prosthesis. The CNN model proves to be the best of three models to be used for knee joint torque estimation. However, future work remains to investigate if this finding extends to an online application with amputees as well. The input of the CNNs were windowed to obtain EMG images, which would introduce a real-time delay of the window size (128 ms) + sampling period (16 ms). Output was filtered by a second order low-pass filter with a cut-off frequency of 1Hz, which would cause an additional real-time delay of 1 sample (16 ms), resulting in a total delay of 160 ms. Therefore, with the intended use in mind, it is essential to test performance of these models in real-time using an actual transfemoral prosthesis, using amputees instead of able-bodied subjects. Futhermore, the sample size of ten subjects is limited and could be extended for a more clear result between the different models. Another limitation of this study is that the influence of the maximum voluntary contractions on the EMG data and model performance have not been investigated. The MVC that was performed for this study cannot be done by a transfemoral amputee. An extension of this study should investigate the use of different MVCs, for instance by using a submaximal contraction to estimate maximum contraction as proposed by Kishimoto et al. (2021) and investigate how different MVCs affect the model performances.

## 5 Conclusion

This study provides new insight into what modelling framework performs best in predicting knee joint torque from EMG data during non-weight bearing activities in healthy subjects. Three models (CNN model, neuromusculoskeletal model and a Hybrid model) were designed and tested on multi-day measurements to gain knowledge about the robustness of each model to time-varying parameters. Results indicate that the CNN model performed best compared to the other models (NRMSE 9.2 ± 4.4%) and that the Hybrid model (NRMSE 12.4 ± 3.4%) was able to outperform the neuromusculoskeletal model (NRMSE 14.3 ± 4.2%). The NMS model was the only model that was robust over all days. The CNN model and Hybrid models showed only significant differences in performance between the first day and all other days, a promising finding for the robustness of each model. These results contribute to the development of a direct control scheme for a transfemoral prosthesis by providing a clear comparison of all three modelling frameworks evualated over multiple days.

## Data Availability

The raw data supporting the conclusions of this article will be made available by the authors, without undue reservation.
